# Identifying the Group Vulnerable to Unmet Medical Needs Due to Food Security: According to Children in the Household

**DOI:** 10.3390/healthcare11030423

**Published:** 2023-02-02

**Authors:** Min-Soo Kim, Hyeon-Ji Lee, Jae-Hyun Kim

**Affiliations:** 1 Institute for Digital Life Convergence, Dankook University, Cheonan 31116, Republic of Korea; 2 Department of Public Health, General Graduate School of Dankook University, Cheonan 31116, Republic of Korea; 3 Department of Health Administration, College of Health Science, Dankook University, Cheonan 31116, Republic of Korea

**Keywords:** food security, unmet medical needs, KNHANES, single parent households, elderly single-person households

## Abstract

Objective: Adults may experience unmet medical needs for various reasons. The purpose of this study was to examine the effect of food security on unmet medical needs according to the presence of children in the household of adults, as well as to identify the medically vulnerable group considering individual and household characteristics. Methods: This study was conducted using data from the National Health and Nutrition Examination Survey for 2013–2015 and 2019–2020. The subjects of the study were 23,069 adults 19 years of age or older, and were divided into two groups according to whether or not children were included in the household. In order to observe the association between food security and unmet medical needs, multiple logistic regression analysis was performed. In addition, a subgroup analysis was performed in consideration of individual and household characteristics. Results: When food security was unstable for households with children, or without children, there was a high correlation with unmet medical needs. Considering individual and household characteristics, in groups with lower age and household income level, or higher number of members in household and subjective health status, food security was strongly correlated with unmet medical needs in households with children. Contrarily, households without children showed a high correlation in the opposite characteristics of households with children, excluding household income level. Conclusion: Food security was highly correlation with unmet medical needs regardless of whether or not children were included in the household. However, according to the individual and household characteristics of households with and without children, the relationship between food security and unmet medical needs was found to be different. Therefore, it is necessary to prepare a health policy that can increase access to medical services in consideration of food security and individual and household characteristics depending on whether or not children are included in the household.

## 1. Introduction

Unmet medical needs can be experienced when there is a discrepancy between the services deemed necessary to solve health problems and the services actually provided [1,2]. Unmet medical needs is used as a simple indicator to monitor the degree of inequality in access and use of medical services [3]. As of 2013, about 3% of the European population reported experiencing unmet medical needs due to cost, waiting time before receiving treatment, and medical services not being available in the region [4], and in 2019, the Organization for Economic Co-operation and Development (OECD) reported a statistic of about 2.6% [5]. In Korea, the medical insurance system was expanded to all citizens in 1989, improving access to medical services [6]. However, the limited scope and level of medical benefits and high co-payments are acting as barriers to access to medical services [7]. From 2007 to 2019, Korea’s unmet medical needs experience rate has been decreasing, but remains high at 11.8% [8], which is high compared to other countries.

The reasons for unmet medical needs can be divided into availability (i.e., waiting time before receiving treatment and timely provision of medical services), accessibility (e.g., cost, transportation expenses, etc.), and acceptability (knowledge and attitude toward medical services, etc.) [9,10]. Among them, the ability to pay medical expenses corresponding to accessibility can act as an important factor in meeting medical demand, especially among people with low socioeconomic status [2,11]. Defining a vulnerable group with a low socioeconomic status is difficult, because the social, economic, cultural, and psychological situations of each individual are different. However, factors that can classify the group as vulnerable include the low income or low education level, as well as unstable employment status [12]. Several previous studies have suggested a link between socioeconomic status and medical use, and economic difficulties are one of the causes of unmet medical needs [6,9,10]. Many people delay or give up treatment due to the financial burden related to medical expenses, and the increase in medical expenses, which accounts for a significant portion of expenditure, results in delays in medical services and treatment that people need [13].

Food security means that everyone has access to sufficient food at all times with the availability of nutritionally appropriate and safe food, as well as the ability to obtain it in a socially acceptable way for an active and healthy life [14]. Conversely, food insecurity is emerging as a global problem. In Canada, 12.7% of the total, which is 1.8 million households or 4.4 million individuals, experienced food insecurity [15]. In addition, 14.5% of households in the United States experienced food insecurity [16], and despite recognizing food insecurity as an important social problem and expanding various social welfare systems to solve the problem, the scale of food insecurity is still over 10.0% [17]. Even in Korea, the occurrence of malnutrition has become extremely rare due to the development of society, but the problem of ensuring food security is deepening due to social polarization [18]. Food security can be a potential risk factor for health [19,20], eating habits [21], chronic diseases [22], and mental stress [23]. Several previous studies have stated that household income level and socioeconomic level including personal income are closely related to food security [19,20,22,24,25,26]. Therefore, unstable food security could mean that the socioeconomic level of an individual or household is low, leading to an inadequate access to necessary medical services, causing inequality in medical use.

Food security may appear differently depending on whether or not children are included in the household. If there are children in the household, parents are burdened with more responsibility for providing food to the children [27], and experience more financial expenditure to buy food [28]. Additionally, households with children have shown lower socioeconomic levels and more unstable food security than those without children [16]. In addition, maintaining the number of positive responses from adults’ survey results regarding food security, households with children expressed concerns about food shortages, and households without children were worried about not being able to eat balanced meals [27]. In other words, it was found that at the same food security level, responses were different depending on the presence or absence of children in the household, emphasizing the necessity to consider whether or not children are included in the household. In previous studies, the relationship between food security and unmet dental care needs [29] as well as medication use [30] was confirmed, and a study examining the relationship between food security and unmet medical needs was conducted on the homeless [31], but there have been a few studies that have divided the study subjects according to the inclusion of children in the household for the general population group. Therefore, this study was conducted to examine the effect of food security on unmet medical needs by dividing adults 19 years of age or older into groups according to whether or not children are included in the household, and to identify the medically vulnerable group considering individual/household characteristics. Through this, we intend to provide empirically basic data to prevent inequality in medical use that may occur in the future and to prepare systematic and efficient support policies.

## 2. Methods

### 2.1. Study Sample and Design

In this cross-sectional study, secondary data analysis was performed using data from the 2013–2015 and 2019–2020 National Health and Nutrition Examination Survey (KNHANES VI, VIII) [32]. The National Health and Nutrition Examination Survey is a legal survey on the health level, health behaviours, and food and nutrition intake for people aged 1 year or older. The Korea Disease Control and Prevention Agency conducts an annual survey and represents the whole country using a two-step stratified sampling method in which the survey area and household are the extraction units. Of the 22,948 survey respondents in 2013–2015 and 15,469 survey respondents in 2019–2020, adults aged 19 years or older were extracted. Among them, respondents who did not respond to the questionnaire on unmet medical needs because they did not use medical services for the past year and those who did not respond to the food security survey were excluded from the study subjects. Respondents who did not respond to other variables included in the study were additionally excluded from the study subjects. Finally, 23,069 study subjects were included in this study. The final study subjects were divided into households with children and households without children using the variable “whether or not children were included in the household”.

### 2.2. Dependent Variables

The dependent variable was unmet medical needs, and the responses to the following questionnaire were used. “During the past year, have you needed medical services (excluding dental treatment), but have you not been able to use them?” Participants responding “yes” were included in the ‘Yes’ group and “no” in the ‘No’ group.

### 2.3. Independent Variables

The independent variable was food security, and the total number of items in the food security survey is composed differently depending on whether children (1 to 18 years old) are included in the household. Households including children are required to respond to a questionnaire consisting of a total of 18 questions including 8 questions related to children, and households without children are required to respond with a total of 10 questions excluding questions related to children. After that, according to the sum of the scores, they were classified into the food security “Security” group (0–2 points) and the food insecurity “Insecurity” group (households with children 3–18 points, households without children 3–10 points).

### 2.4. Control Variables

The following variables were included in the analysis as covariates according to the Anderson model. Predisposing factors (i.e., gender, age, education level), enabling factors (i.e., household income level, residency area, number of household members, children included in the household), and need factors (i.e., current smoking status, monthly alcohol drinking status, strength exercise practice rate, subjective health status, number of chronic diseases, inpatient use in the past year, outpatient use in the past 2 weeks). In addition, strength exercise practice rate was categorized as ‘Yes’ or ‘No’ as the percentage of strength exercise such as push up, sit up, dumbbell, weightlifting, and iron bars was being practiced for 2 days or more in the past week.

### 2.5. Analytical Approach and Statistics

The chi-square test was used to compare the differences in the distribution of the dependent variable according to the independent variable. Logistic regression analysis including covariates was performed to investigate the association between food security and unmet medical needs. A subgroup analysis was performed to examine the effect between food insecurity and unmet medical needs according to individual and household characteristics. All analyses were conducted by dividing households with children and households without children, and weights recommended by the National Health and Nutrition Examination Survey (KNHANES) were applied. Statistical significance was set at *p* < 0.05, and association was presented as odds ratio (OR) and *p*-value. All statistical analyses were performed using the SAS statistical software package version 9.4 (SAS Institute Inc., Cary, NC, USA).

## 3. Results

### 3.1. Sample Characteristics

Table 1 shows the results of comparing the sociodemographic characteristics of the study subjects. Of the total of 23,069 people, 2455 (10.6%) reported that they had unmet medical needs. Of the 21,490 people who were in the ‘Security’ group, 2069 people (9.6%) answered that they had unmet medical needs, and among 1579 people who were in the ‘Insecurity’ group, 386 people (24.4%) responded that they had unmet medical needs, indicating that the people in the ‘Insecurity’ group experienced unmet medical needs at a higher rate than the group who were in the ‘Security’ group. This trend of higher rate of unmet medical needs was also apparent when the subjects were divided into households with or without children.

### 3.2. Adjusted Effect of Food Security and Unmet Medical Needs

The association between food security and unmet medical needs is shown in Table 2. In the total population, the odds ratio (OR) for the unmet medical needs in the ‘Insecurity group was 1.92 (*p* < 0.0001) with the ‘Security’ group as reference. According to age, the ORs of the ‘40–59 years old’ and ‘60 years old’ group were 0.79 (*p* = 0.001) and 0.66 (*p* < 0.0001), respectively, compared to the ‘19–39 years old’ group. As for subjective health status, the OR of the ‘Bad’ group was 3.89 (*p* < 0.0001) compared to the ‘Good’ group. When all study subjects were divided into households with children or households without children, the OR for the unmet medical needs of ‘Insecurity’ group compared to the ‘Security’ group for food security was 1.97 (*p* < 0.0001) for household with children, and 2.00 (*p* < 0.0001) for household without children. Compared to the ‘19–39’ group, the OR of the ‘40–59 years old’ and ‘60 years old or older’ groups was 0.74 (*p* = 0.004) and 0.66 (*p* = 0.050) for household with children, and 0.83 (*p* = 0.057) and 0.67 (*p* = 0.001) for household without children. As for subjective health, compared to the ‘Good’ group, the OR of the ‘Bad’ group was 4.91 (*p* < 0.0001) for households with children and 3.38 (*p* < 0.0001) for households without children, indicating that the ORs for unmet medical needs of households with children were higher.

### 3.3. The Relationship between Food Insecurity and Unmet Medical Care Considering Individual and Household Characteristics

Table 3 shows the results of analysis of the relationship between food insecurity and unmet medical needs depending on age, number of household members, household income level, and subjective health status. When all households were divided into households with children and households without children, the OR of unmet medical needs for food insecurity was 2.07 (*p* = 0.004) for ‘19–39 years old’ in households with children and increased as the age decreased. However, the OR was the highest in the ’60 years old or older’ group (OR: 2.47, *p* < 0.0001) in households without children. Additionally, in households with children, when the number of household members was ‘three or more’, the OR was 1.98 (*p* < 0.0001), which was higher as the number of household members increased. In households without children, the OR of unmet medical needs was higher as the number of household members decreased (‘1’; OR = 2.34, *p* = 0.031). In terms of the household income levels, the OR of unmet medical needs was 2.78 (*p* = 0.001) and 2.42 (*p* < 0.0001), respectively, in the ‘Low’ group for both households with and without children. The OR of unmet medical needs was higher as the subjective health status improved in the households with children (‘Good’; OR = 3.48, *p* = 0.001), but in the households without children, the OR of unmet medical needs was higher as the subjective health worsened (‘Bad’; OR = 2.38, *p* < 0.0001).

## 4. Discussion

In order to improve access to medical services for adults over the age of 19, this study observed the association between food security and unmet medical needs, considering the inclusion of children in the household. Furthermore, by examining the relationship between food security and unmet medical needs in consideration of individual and household characteristics, it aimed to identify the medically vulnerable group to reduce medical inequality. The results of our study presented an association between food insecurity and unmet medical needs, and this association remained significant in both households with children and without children. When considering individual characteristics such as age, subjective health status, and household characteristics such as number of household members and household income level, the relationship between food insecurity and unmet medical needs was different. According to the results of this study, possible discussions are as follows.

Regardless of whether children were included in the household, households with food insecurity experienced a higher rate of unmet medical needs than households with food security. According to our findings, people in the ‘Insecurity’ group experienced unmet medical needs both in households with children (22.1%) and without children (25.8%), which were higher than the previously reported unmet medical needs of Greece [2] and Canada [33], as well as Koreans aged 65 years or older [6]. These results are similar to those of studies examining the relationship between food shortages and unmet medical needs for the homeless [31], and studies showing that food insecurity affects unmet dental care needs [29], but there is a need to reconfirm the relationship between food security and unmet medical needs through further follow-up studies in the general population. In addition, younger participants were more likely to experience unmet medical needs, and this association could be explained by the lack of knowledge about medical service resources, evaluation of various symptoms, and inappropriate use of medical care [34]. Furthermore, poorer subjective health status was associated with higher likelihood of experiencing unmet medical needs, indicating that people who require more medical services are not able to receive the necessary services [11]. Therefore, in order to improve access to unmet medical needs such as medical service inequality, it is necessary to consider individual characteristics such as age and subjective health status as well as food security.

According to the individual and household characteristics of households with and without children, the relationship between food security and unmet medical needs was found to be different. In the case of households with children, lower age, higher number of household members, lower household income level, and better subjective health status were associated with higher unmet medical needs due to food insecurity. In the case of a young age, the socioeconomic status is relatively low [33], and a low socioeconomic level may lead to more unstable food security [16]. Considering how households with children pay more for food than households without children [28], an association between higher number of household members [23], and lower the household income [19,35], with more unstable the food security as well as unmet medical needs can be assumed. In addition, the characteristics of younger adults to evaluate subjective health status more positively [36], could have affected the relationship between high food insecurity and unmet medical needs despite the good subjective health status. Although there was no statistical significance in the case of two household members in a household with children, the association between food insecurity and unmet medical needs was high. These results are expected to occur in single-parent households composed of children and their guardians. The poverty rate related to food security was highest in single-parent households [37,38], and it can be said that they have a high risk of food insecurity [39]. In fact, 31% of all single-parent households in the United States reported experiencing food insecurity [17], and the group with the highest prevalence of food insecurity was single-parent households [37,40]. The possibility of food insecurity was more than three times higher in single-parent households with children and no spouses than in married households with children [19], in this study, there was a high correlation between food insecurity and unmet medical needs in households with two members. In the case of households without children, subjects with higher age, smaller number of household members, lower household income level, and worse the subjective health status, stronger association of unmet medical needs due to food insecurity was demonstrated. A possible assumption for the people with these characteristics is that they are a group of elderly single-person households without children with low household income level and poor subjective health status with unstable food security and high possibility of experiencing unmet medical needs. In fact, the elderly living alone may be the most vulnerable to food insecurity [41], and in Korea and the United States, most of the people with food insecurity were living in single-person households [42]. Living alone can negatively affect the elderly’s access to food and the adequacy of food consumption in many ways [41]. In addition, as with households with children, lower household income was associated with more unstable the food security [19,39], Subjective health status was evaluated negatively as the age increased [36], affecting the correlation between food insecurity and unmet medical needs to be high. Therefore, if food security is secured for adults, there is a possibility of reducing unmet medical needs, but before implementing a food support policy, there is a need to implement a support policy tailored to each household taking into account the individual and household characteristics of households with and without children.

By verifying the association between food security and unmet medical needs, this study confirmed that when food security is unstable, unmet medical needs can also be experienced, and that food security can be used as a variable to discover medically vulnerable groups. In addition, the study was conducted by dividing the study subjects into households with children and households without children, and sub-group analysis was conducted considering individual and household characteristics to examine the relationship between food insecurity and unmet medical needs according to each characteristic level. The representativeness of the study subjects was secured by conducting an analysis considering weights using national sample data. The results of this study can be used as basic data to support health policy establishment to improve access to medical services and identify medically vulnerable groups by exploring the causes of unmet medical needs in adults in terms of food security.

There are several limitations to be aware of when interpreting the results of this study. In the questionnaire used in the study, food security and unmet medical needs were measured with a self-reported questionnaire, so there may be bias among respondents. In the data, the evaluation criteria for food security were calculated differently depending on whether or not children were included in the household. Because the responses of households may differ depending on the level of food security [27], we endeavoured to compensate for this limitation by dividing the study subjects into households with children and households without children. In addition, unmet medical needs may be different from the clinical approach because it is a single-item response based on individual subjective judgment. However, this concept was used due to the unique characteristic of being able to measure the shortcomings of the medical service experience [43]. Lastly, since it is a cross-sectional study, there may be limitations in interpreting the study results as a causal relationship.

## 5. Conclusions

Regardless of whether or not children were included in the household, when food security was unstable, the association with unmet medical needs was higher. In addition, the association of unmet medical needs due to food insecurity was high in households with children, households with three or more members, and in elderly households without children with one person. In future studies, household types should be subdivided into single-parent households, multi-person households, and elderly households living alone to further examine the relationship between food security and unmet medical needs in more detail. State and community support is provided for nutritional and medical deficiencies. However, for efficient support, it is necessary to prepare health policies that can increase access to medical services and reduce medical inequality in consideration of food security, individual and household characteristics, depending on whether or not children are included in the household.

## Figures and Tables

**Table 1:** General characteristics of participants at baseline.

Variables		Unmet Medical Needs										
		Total Household					Household with Children			Household without Children		
		Total		Yes		*p*-Value ^a^	Yes		*p*-Value ^a^	Yes		*p*-Value ^a^
		N	% *	N	% *		N	% *		N	% *	
**Food security**						<0.0001			<0.0001			<0.0001
	Security	21,490	93.2	2069	9.6		655	9.6		1414	9.6	
	Insecurity	1579	6.8	386	24.4		130	22.1		256	25.8	
**Gender**						<0.0001			<0.0001			<0.0001
	Male	9696	42.0	719	7.4		217	7.6		502	7.4	
	Female	13,373	58.0	1736	13.0		568	12.5		1168	13.2	
**Age**						0.0534			0.0002			0.9826
	19–39	6364	27.6	708	11.1		343	11.8		365	10.6	
	40–59	8585	37.2	852	9.9		345	9.2		507	10.5	
	60–	8120	35.2	895	11.0		97	13.1		798	10.8	
**Residency region**						0.8096			0.9441			0.7364
	Metropolitan	10,501	45.5	1132	10.8		362	11.1		770	10.6	
	Else	12,568	54.5	1323	10.5		423	10.2		900	10.7	
**Educational level**						<0.0001			0.0003			<0.0001
	Middleschoolorless	7350	31.9	1020	13.9		147	15.7		873	13.6	
	Highschool	7737	33.5	728	9.4		272	9.9		456	9.1	
	Collegeorover	7982	34.6	707	8.9		366	9.8		341	8.0	
**Household income level**						<0.0001			0.1336			<0.0001
	Low	4347	18.8	678	15.6		90	15.4		588	15.6	
	Middle-low	5782	25.1	623	10.8		213	11.0		410	10.7	
	Middle-high	6244	27.1	593	9.5		254	10.1		339	9.1	
	High	6696	29.0	561	8.4		228	9.6		333	7.7	
**No. household members**						0.0002			0.3587			0.0002
	1	2549	11.0	375	14.7		0	0.0		375	14.7	
	2	7053	30.6	708	10.0		16	9.4		692	10.1	
	3–	13,467	58.4	1372	10.2		769	10.6		603	9.7	
**Children included in the household**						0.7707						
	No	15,653	67.9	1670	10.7		-	-		1670	10.7	
	Yes	7416	32.1	785	10.6		785	10.6		-	-	
**Current smoking status**						0.0394			0.4928			0.0404
	No	19,156	83.0	2021	10.6		639	10.5		1382	10.6	
	Yes	3913	17.0	434	11.1		146	10.8		288	11.2	
**Monthly alcohol drinking status**						0.0002			0.0033			0.0098
	No	11,074	48.0	1330	12.0		376	12.2		954	11.9	
	Yes	11,995	52.0	1125	9.4		409	9.4		716	9.4	
**Strength exercise practice rate**						<0.0001			0.0636			<0.0001
	No	18,270	79.2	2057	11.3		652	10.8		1405	11.5	
	Yes	4799	20.8	398	8.3		133	9.5		265	7.8	
**Subjective health status**						<0.0001			<0.0001			<0.0001
	Good	6898	29.9	396	5.7		133	5.3		263	6.0	
	Normal	11,745	50.9	1126	9.6		411	10.6		715	9.1	
	Bad	4426	19.2	933	21.1		241	23.3		692	20.4	
**No. chronic diseases**						<0.0001			0.0005			0.0021
	0	12,538	54.3	1226	9.8		521	9.8		705	9.8	
	1	4968	21.5	519	10.4		152	11.6		367	10.0	
	2	2888	12.5	325	11.3		58	12.8		267	11.0	
	3–	2675	11.6	385	14.4		54	16.9		331	14.1	
**Inpatient use in the past year**						0.0001			0.1531			0.0003
	No	20,372	88.3	2099	10.3		692	10.4		1407	10.3	
	Yes	2697	11.7	356	13.2		93	12.3		263	13.6	
**Outpatient use in the past 2 weeks**						<0.0001			<0.0001			0.0602
	No	15,295	66.3	1509	9.9		511	9.6		998	10.0	
	Yes	7774	33.7	946	12.2		274	13.2		672	11.8	
**Year**						<0.0001			<0.0001			<0.0001
	2013	4936	21.4	644	13.0		225	11.8		419	13.9	
	2014	4379	19.0	523	11.9		206	14.0		317	10.9	
	2015	4672	20.3	596	12.8		163	11.3		433	13.4	
	2019	5053	21.9	384	7.6		106	7.1		278	7.8	
	2020	4029	17.5	308	7.6		85	7.7		223	7.6	
**Total**		23,069	100.0	2455	10.6		785	10.6		1670	10.7	

*: Weighted; ^a^: Rao Scott chi square test.

**Table 2:** Adjusted effect of food stability and unmet medical needs.

Variables		Unmet Medical Needs *					
		Total Household		Household with Children		Household without Children	
		OR	*p*-Value	OR	*p*-Value	OR	*p*-Value
**Food security**							
	Security	1.00		1.00		1.00	
	Insecurity	1.92	<0.0001	1.97	<0.0001	2.00	<0.0001
**Gender**							
	Male	1.00		1.00		1.00	
	Female	1.78	<0.0001	1.74	<0.0001	1.85	<0.0001
**Age**							
	19–39	1.00		1.00		1.00	
	40–59	0.79	0.001	0.74	0.004	0.83	0.057
	60–	0.66	<0.0001	0.66	0.050	0.67	0.001
**Residency region**							
	Metropolitan	1.00	0.945	0.98	0.822	1.03	0.709
	Else	1.00		1.00		1.00	
**Educational level**							
	Middleschoolorless	1.13	0.155	0.98	0.922	1.21	0.066
	Highschool	0.92	0.192	0.80	0.027	1.03	0.751
	Collegeorover	1.00		1.00		1.00	
**Household income level**							
	Low	1.24	0.022	0.82	0.249	1.49	0.001
	Middle-low	1.09	0.281	0.88	0.305	1.26	0.028
	Middle-high	1.05	0.519	0.97	0.779	1.10	0.367
	High	1.00		1.00		1.00	
**No. household members**							
	1	1.05	0.606			1.02	0.816
	2	0.93	0.330	0.58	0.086	0.95	0.457
	3–	1.00		1.00		1.00	
**Children included in the household**							
	No	1.00					
	Yes	0.96	0.564				
**Current smoking status**							
	No	1.00		1.00		1.00	
	Yes	1.37	<0.0001	1.32	0.051	1.41	0.000
**Monthly alcohol drinking status**							
	No	1.00		1.00		1.00	
	Yes	1.01	0.918	0.92	0.358	1.06	0.465
**Strength exercise practice rate**							
	No	1.04	0.561	0.89	0.332	1.12	0.200
	Yes	1.00		1.00		1.00	
**Subjective health status**							
	Good	1.00		1.00		1.00	
	Normal	1.69	<0.0001	2.05	<0.0001	1.49	<0.0001
	Bad	3.89	<0.0001	4.91	<0.0001	3.38	<0.0001
**No. chronic diseases**							
	0	1.00		1.00		1.00	
	1	1.00	0.982	1.13	0.344	0.92	0.410
	2	0.96	0.655	1.24	0.307	0.85	0.133
	3-	1.03	0.774	1.31	0.289	0.94	0.582
**Inpatient use in the past year**							
	No	1.00		1.00		1.00	
	Yes	1.02	0.828	0.95	0.737	1.06	0.518
**Outpatient use in the past 2 weeks**							
	No	1.00		1.00		1.00	
	Yes	1.00	0.954	1.18	0.101	0.93	0.258
**Year**							
	2013	1.00		1.00		1.00	
	2014	0.94	0.414	1.14	0.336	0.83	0.066
	2015	1.01	0.949	0.86	0.262	1.07	0.510
	2019	0.57	<0.0001	0.60	0.001	0.55	<0.0001
	2020	0.57	<0.0001	0.59	0.002	0.56	<0.0001

*: Weighted.

**Table 3:** Age and number of household members, household income level, subjective health status specific association of unmet medical needs.

Variables		Unmet Medical Needs *								
		Total Household			Household with Children			Household without Children		
		Security	Insecurity		Security	Insecurity		Security	Insecurity	
		OR	OR	*p*-Value	OR	OR	*p*-Value	OR	OR	*p*-Value
**Age**										
	19–39	1.00	1.55	0.014	1.00	2.07	0.004	1.00	1.34	0.246
	40–59	1.00	2.01	<0.0001	1.00	2.07	0.001	1.00	2.07	<0.0001
	60–	1.00	2.34	<0.0001	1.00	1.86	0.077	1.00	2.47	<0.0001
**No. household members**										
	1	1.00	2.34	<0.0001		-	-	1.00	2.34	<0.0001
	2	1.00	2.12	<0.0001	1.00	2.91	0.197	1.00	2.15	<0.0001
	3–	1.00	1.74	<0.0001	1.00	1.98	<0.0001	1.00	1.53	0.031
**Household income level**										
	Low	1.00	2.36	<0.0001	1.00	2.78	0.001	1.00	2.42	<0.0001
	Middle-low	1.00	2.12	<0.0001	1.00	2.35	<0.0001	1.00	1.97	0.001
	Middle-high	1.00	1.55	0.033	1.00	1.70	0.083	1.00	1.43	0.179
	High	1.00	1.08	0.880	1.00	1.01	0.991	1.00	1.10	0.880
**Subjective health status**										
	Good	1.00	1.85	0.007	1.00	3.48	0.001	1.00	1.37	0.291
	Normal	1.00	1.76	<0.0001	1.00	1.81	0.002	1.00	1.83	0.000
	Bad	1.00	2.12	<0.0001	1.00	1.83	0.023	1.00	2.38	<0.0001

*: Weighted; Control variables: Gender, Age, Residency region, Educational level, Household income level, No. household members, Children included in the household, Current smoking status, Monthly alcohol drinking status, Strength exercise practice rate, Subjective health status, No. chronic diseases, Inpatient use in the past year, Outpatient use in the past 2 weeks, Year.

## Data Availability

The data that support the findings of this study are openly available in the National Health and Nutrition Examination Survey at https://knhanes.kdca.go.kr/.
